# Targeting in Cancer Therapies

**DOI:** 10.3390/medsci4010006

**Published:** 2016-03-08

**Authors:** Ramon Mangues, Esther Vázquez, Antonio Villaverde

**Affiliations:** 1 Biomedical Research Institute Sant Pau (IIB-SantPau) and Josep Carreras Leukemia Research Institute, Hospital de la Santa Creu i Sant Pau, 08025 Barcelona, Spain; rmangues@santpau.cat (R.M.); Esther.Vazquez@uab.es (E.V.); 2 CIBER de Bioingeniería, Biomateriales y Nanomedicina (CIBER-BBN), Bellaterra, 08193 Cerdanyola del Vallès, Spain; 3 Institut de Biotecnologia i de Biomedicina, Universitat Autònoma de Barcelona, Bellaterra, 08193 Cerdanyola del Vallès, Spain; 4 Departament de Genètica i de Microbiologia, Universitat Autònoma de Barcelona, Bellaterra, 08193 Cerdanyola del Vallès, Spain

Drug developers recruit and combine principles, procedures and strategies from chemistry, pharmacology, nanotechnology and biotechnology, focusing on the generation of functional vehicles as nano-carriers of drugs for improved stability and enhanced intracellular delivery. By exploring macromolecules such as lipids, polymers and proteins, both biocompatibility and cost-effective fabrication are favored, and a plethora of opportunities for architectonic and functional adaptation of vehicles to particular cargo drugs and therapeutic conditions enlighten the cancer treatment scenario. Molecular vehicles specifically developed as carriers for drugs are usually referred to as drug delivery systems (DDS). The application of nanoscale technologies has resulted in an already vast but still expanding catalogue of drugs and appropriate DDS, whose implementation in therapy is heavily represented in the different phases of drug development, from basic research to clinical trials [1]. DDS, as a conceptual but also structural platform, allows decreasing dose frequency and reduce drug fluctuation in the bloodstream [2]. The nanostructured drugs for cancer treatment that have so far reached the oncology market use passive targeting (p.e.: Abraxane, Doxil, Daunoxome, Oncaspar, DepoCyt), meaning that they are not empowered by specific mechanisms to recognize specific cell types or tissues. Preferential but still passive accumulation into tumor tissues is then favored; the plain increase in circulation time is promoted by the nanoscale size of the drug-vehicle conjugate and the tumor’s enhanced permeability and retention effect (EPR) [3]. The amount of drug that reaches target cells is supposed to remain low [4] and insufficient to ensure a maximal reduction of undesired drug side effects due to its activity over healthy tissues. In fact, this is a moderate advance regarding conventional cancer chemotherapy since current anticancer drugs display a low therapeutic index. Most of such conventional chemotherapeutic drugs (e.g., 5-fluorouracil) exert their antitumor effect by interfering with nucleic acid synthesis and inhibiting tumor cell proliferation. When the level of DNA damage in exposed, cells exceed their repair capacity, and induction of cell death follows. Their low molecular weight allows their free diffusion through the body, so that they also reach normal tissues. Their greatest effect occurs, however, in highly proliferative normal cells (*i.e.*, bone marrow and intestinal tract), often causing dose-limiting myelosuppression and gastrointestinal toxicities. The conjunction of this narrow therapeutic index and the considerable inter-individual differences in distribution, metabolism and excretion of these cytotoxic agents in humans, results in increased risks of toxicity and sub-therapeutic dosing in the individual patient [5].

This landscape may soon change since many actively targeted nanoparticles for drug delivery are being evaluated in clinical assays [6]. Among them, a doxorubicin-loaded anti-EGFR immunoliposome for the treatment of different EGFR-overexpressing cancers and a PLA-PEG nanoparticle that targets docetaxel to PMSA^+^ prostate cancer cells are representative examples [7]. We expect that cell-targeted nanoparticles further improve the benefits already achieved by passively targeted nanoparticles, in a similar manner to the increased selectivity in tumor uptake and antitumor effect already achieved by the cell-targeted antibody-drug conjugates (ADCs). Development of ADCs has allowed the incorporation of more potent toxins (100–1000 times more cytotoxic than classical chemotherapeutics) instead of conventional drugs as payloads. Two of these ADCs have already reached the market: Trastuzumab Emtamsine, target to HER2/neu receptor for breast cancer treatment [8], and Brentuximab Vedotin, targeted to the surface protein CD30, for Hodgkin lymphoma (HL) and systemic anaplastic large cell lymphoma (sALCL) treatment [9]. Currently, there are another 30 ADCs in clinical trials, indicative of the expectations regarding controlled drug delivery and also proving the strength and robustness of the targeting approaches represented by ADCs in the development of novel and innovative cancer medicines.
